# Health Tract, No. 156

**Published:** 1865

**Authors:** 


### HEALTH TRACT, No. 156.

### SABBATH OBSERVANCE.

The nations of the earth which now most respect the Sabbath, and most discourage labor, pastimes, and mere amusements, during its sacred hours, are the freest, the happiest, the most prosperous, and the farthest advanced in the progress of art, manufacture, and invention; and that city or town or village or community, of any Sabbath-respecting nation, which best keeps the Sabbath as a day of rest for body and mind, is the most noted for all that is orderly, law-abiding, and substantial; and that family, of any Sabbath-loving community, which best observes it by quiet, by religious worship, and the performance of Bible duties, is the most substantial and respected and reliable in that community, while any individual member of a Sabbath-keeping family who most spends the hours of that sacred day in meditation, in worship, and the prayerful reading of the Scriptures, will uniformly be found to follow a blameless life; to possess the respect and confidence of the whole community; and all men will know where to look for him, however evil may be the times—to wit, on the side of justice and right and liberty and law and sterling principle.

No man can be so blinded as not to know that the Sabbath is least respected where there is most of all that is vulgar and profane and abandoned; and that those who care the least for it are literally thieves and murderers, drunkards, prize-fighters, horse-racers, and the utterly depraved of all classes; and that these, the wicked, do "not live out half their days." As a means then of longevity, of worldly prosperity, of individual elevation of character, every good citizen will not only do what is possible in himself to secure a religious observance of the Sabbath-day, will not only countenance and encourage others to do the same, but will *volunteer* his pecuniary aid to further these things in the community around him.

For some years past a number of gentlemen of eminence, socially, civilly, and financially, have, as "The New-York Sabbath Committee," been laboring with extraordinary steadiness of purpose, dignity, wisdom, and success, for the promotion of the better observance of the Sabbath-day in the metropolis of the nation. In doing this they have labored day and night; have encountered innumerable obstacles; have met with every variety of discouragement, obloquy, and opposition from all classes of society, except the wisest, the highest, and the best; and without bluster, without threats, without vituperation, abuse, or epithet, but by the calm, dignified, and persistent presentation of indisputable facts and sterling principles, they have gone on from step to step, "conquering and to conquer;" have put down the crying of Sunday papers; have abolished the open and shameless Sunday liquor-traffic; have driven the concert-saloons out of existence; and with these plague-spots have passed away the atheistic advocates of "The People's Day," "The Poor Man's Day," "Sunday Theaters," "No Sunday at All," and "Sunday Rum." In doing these things they have printed and widely circulated twenty-four "Sabbath Documents," from eight to thirty-two octavo pages each, in beautiful large print, containing a vast amount of Sabbath literature, which Christians of all countries would be delighted to read. But let it be remembered by every reader that a limited number of gentlemen have sustained this movement from the outset, without appealing to the general public for funds. The enterprises now in progress contemplate *national* reforms, involving increased expenditures. Where is the reader who will not desire to participate in the pleasure of promoting them, and promptly forward his liberal and free-hearted contribution to J. M. Morrison, Esq., President of the Manhattan Bank, New-York City, who is Treasurer? Letters and orders for "Sabbath Documents" may be addressed to "The Secretary of the Sabbath Committee, No. 5 Bible House, New-York."

